# Two-Stage CNN Model for Joint Demosaicing and Denoising of Burst Bayer Images

**DOI:** 10.1155/2022/6200931

**Published:** 2022-04-04

**Authors:** Hanlin Tan, Huaxin Xiao, Yu Liu, Maojun Zhang

**Affiliations:** College of System Engineering, National University of Defense Technology, Changsha 410073, China

## Abstract

In the classical image processing pipeline, demosaicing and denoising are separated steps that may interfere with each other. Joint demosaicing and denoising utilizes the shared image prior information to guide the image recovery process. It is expected to have better performance by the joint optimization of the two problems. Besides, learning recovered images from burst (continuous exposure images) can further improve image details. This article proposes a two-stage convolutional neural network model for joint demosaicing and denoising of burst Bayer images. The proposed CNN model consists of a single-frame joint demosaicing and denoising module, a multiframe denoising module, and an optional noise estimation module. It requires a two-stage training scheme to ensure that the model converges to a good solution. Experiments on multiframe Bayer images with simulated Gaussian noise show that the proposed method has obvious performance advantages and speed advantages compared with similar approaches. Experiments on actual multiframe Bayer images verify the denoising effect and detail retention ability of the proposed method.

## 1. Introduction

A digital camera usually captures a raw image and uses an image processing pipeline to output a full-color image. The raw image is a digital matrix captured by a camera sensor and determined by a color filter array (CFA) on top of the sensor. Each pixel location of CFA consists of only one color among red, green, and blue. Therefore, an interpolation process called demosaicing is required to recover the full-color image with three color channels. Besides, the captured raw image is contaminated with noise. Therefore, a denoising step is also required. As a result, demosaicing and denoising are two separate steps that contribute to the output of a clean full-color image in a traditional image processing pipeline.

The major drawback of separating demosaicing and denoising is that they interfere with each other. If demosaicing is performed first, the noise distribution is changed by the interpolation process, which makes it harder for denoising to remove noise. If denoising is performed first, color samples in the raw image are changed, which makes it more difficult for demosaicing to recover full colors.

Recovery of the full-color clean image from the noisy raw image is an ill-posed problem. Prior knowledge about image statistics, or image priors, is required to constrain the solution space of the problem to get reasonable results. Demosaicing and denoising can be jointly performed based on the same image priors [1–7], which comes from three aspects: (a) the image priors can be manually designed. Condat et al. [8] use total variation (TV) prior to ensure the smooth property of image in joint demosaicing and denoising. Heide et al. [9] propose a minimization model that combines TV priors with BM3D and cross-channel priors to improve the quality of the recovered image. Park et al. [10] introduce a convolutional neural network (CNN) model as prior to further improve image details. (b) The image priors can be learned from the image dataset. Khashbi et al. [2] uses random fields to fit the problem of joint demosaicing and denoising. Klatzer et al. [3] model the problem as a minimization problem and learn from the image dataset to improve performance. Khashabi et al. [2] introduce regression tree fields to learn image datasets through a specific loss function. Gharbi et al. [1] design a deep convolutional neural network (CNN) model for joint demosaicing and denoising, which firstly introduce CNN to solve the problem. Liu et al. [6] propose a density-map guidance to help the model deal with a wide range of frequencies, which improves recovered image quality. Xing et al. [7] carefully study the CNN model structure and loss functions to further improve recovered image quality. (c) The image priors can be extracted from multiple frames of the same scene or burst photography. Kokkinos et al. [4] propose an iterative framework to optimize a burst of raw images separately processed by Gharbi's CNN model. The method combines the CNN model with burst photography for joint demosaicing and denoising, which improves recovered image quality.

However, there are some drawbacks to Kokkinos's method. First, the CNN model and the iterative framework are not jointly optimized. It means when they optimize the results from image bursts using the iterative framework, the weights of the CNN model are fixed. A natural idea is to jointly optimize the two separate steps and further improve recovery performance. Second, the iterative framework is slow in deployment. If the burst input can be processed by a single CNN model without the iterative process, the running speed in deployment can be significantly improved.

**Figure 1:**
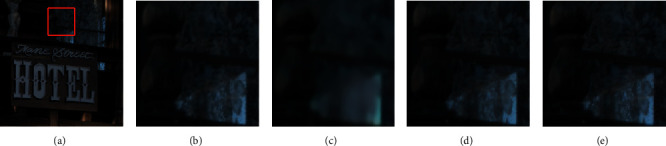
Demosaicing and denoising results on real burst. (a) A Scene. (b) Interp.+V-BM4D. (c) BDNet [1]. (d) M2M [2]. (e) TwoStage (Ours).

In this article, we propose a unified CNN model to solve the problem of joint demosaicing and denoising of burst images. The model contains three submodules to process a single image [11], multiple frames [12], and noise estimation [13]. With a carefully designed network architecture and a two-stage training strategy, the proposed model outperforms comparative methods in both recovery performance and processing speed.  Figure 1 shows a comparison of burst demosaicing and denoising methods on a real burst, which will be further explained in Subsection 3.4.

## 2. Method

### 2.1. Problem Formation

Given noisy multiframe raw images (burst images) of the same scene, the goal of joint demosaicing and denoising is to generate a noise-free and clear linear RGB image corresponding to the scene. Using the image redundancy information in multiple frames, joint demosaicing and denoising of burst images may achieve better image quality than single-frame demosaicing and denoising does.

Suppose **b** to be a collection of noisy raw images continuously exposed by multiple frames of the same scene, and *y* is the noise-free linear RGB image of the scene corresponding to the reference frame in **b**. We construct a training dataset {(**b**_*i*_, *y*_*i*_)*|i*=1,…, *M*} in multiple scenarios and learn the joint demosaicing and denoising mapping *F*(**b**; *θ*) so that

(1) $$\begin{array}{c} \min_{\theta }\sum_{i}{\left\|F\left(dm\left(b_{i}\right);\theta \right)-y_{i}\right\|}_{1}, \end{array}$$

where *θ* denotes the model parameters and *F*(**dm**(·); *θ*) is a multiframe joint demosaicing and denoising function implemented by a CNN, in which **dm** represents a demosaicing function included in the CNN. We use *l*_1_-norm rather than *l*_2_-norm here to suppress blurry effects.

The above formalization process reflects the main idea of dealing with joint demosaicing and denoising of burst images: first, use the network module **dm** to jointly demosaic and denoise each image to obtain a linear RGB image, and then use the network module *F* that performs multiframe denoising on the obtained multiframe linear RGB images. In this process, each frame of the input image undergoes single-frame and multiframe denoising in two stages, contributing to the final clear linear RGB image.

### 2.2. Network Design

The multiframe joint demosaicing and denoising network is mainly composed of two existing major modules: the one is a single-frame joint demosaicing and denoising module, which is implemented using the DRDD [11] network structure. It consists of a series of residual blocks that learn a joint demosaicing and denoising mapping directly. The DRDD network has several designs to improve image recovery performance [11]. First, it splits pixels of Bayer images into three color channels as input. In contrast, directly input Bayer images to CNN will lead to a significant performance drop. Second, it studies the influence of residual blocks and it proves to be effective. Third, it introduces a noise level map at every residual block to strengthen noise information in the deep part of CNN and turns out to be beneficial.

The other is a multiframe denoising module, which adopts the multiframe denoising network structure MF-SE-DRDD [12]. It performs end-to-end denoising of a burst of images. The input of the module is a burst of initially denoised RGB images, which goes through a stack of residual blocks followed by a convolution and ReLu [14] activation. It first outputs *n* intermediate clean estimates, then uses a squeeze-and-excitation (SE) module to get those estimates channel-wise weighted, and at last uses simple element-wise addition to merging the final clean output, which will be further explained in Section 2.4. Besides, the noise level map required for denoising can be estimated by the noise estimation module [13], or it can be input together with the multiframe noise level map.

**Figure 2:**
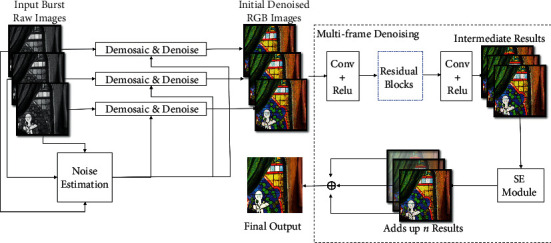
Network architecture for joint demosaicing and denoising of burst images.

The overall network structure of the multiframe joint demosaicing and denoising network is shown in Figure 2. First, suppose we have *n* input frames and the *n*-frame raw images go through the joint demosaicing and denoising module, respectively, and output the initial denoising multiframe linear RGB images. In the figure, multiple joint demosaicing and denoising modules share weights. This step performs demosaicing and first-level denoising in a single-frame image. Then, the initial multiframe linear RGB images are concatenated into a 3*n*-channel tensor and input to the multiframe denoising module. In the multiframe denoising module, the input tensor passes through a series of residual blocks to obtain an intermediate result aligned to the reference frame, then goes through the squeeze-and-excitation (SE) module, and adds up to obtain the final linear RGB image. In this step, multiple frames of redundant information are used for the second level of noise removal. The ablation experiment in Section 3.5 shows that this two-stage denoising design can remove the noise in the image more effectively when the noise level is large.

In the traditional methods of computer vision, raw image demosaicing is usually solved by interpolation; the problem of multiframe denoising usually requires multiple frames to be aligned and then weighted and averaged; these two methods have great differences. In the trial stage of the network design, we have tried to complete these two steps in the same network, but the result was poor. The reason is that a slight error in multiframe alignment will cause significant color disorder in the raw image interpolation. Therefore, it is extremely difficult to learn to solve these two problems at the same time. In this article, we use two subnetwork structures to solve these two problems, which reduces the difficulty of the entire problem.

### 2.3. Synthetic Training Data

Training data are essential for joint demosaicing and denoising performance. Since it is difficult to get real bursts with groundtruth [15], we have to synthetic training data mainly in two steps. First, simulate clean Bayer images from clean RGB images as described in [12], and add a specific type of noise to Bayer images and obtain clean and noisy image pairs. Then, we need to design a frame displacement model to simulate displacement in real bursts.

#### 2.3.1. Generating Clean and Noisy Image Pairs

Groundtruth training images are required to be clean and rich textured. We select the first 4,000 images from the Waterloo exploration dataset [16] to construct training data. Images are cropped into 128 × 128 nonoverlapping patches with a stride of 256. The rest images from the Waterloo exploration dataset are used as validation data to select a good model weight.

We train two types of models with different additive noise: first, white Gaussian noise for easy quantitative comparison with previous methods; simulating white Gaussian noise is quite easy since we only have to generator random values subject to Gaussian distribution with a given noise level; and second, simulated real noise for qualitative comparison on real noisy bursts.

Noise in raw images can be well modeled by a Poisson Gaussian distribution [17]:

(2) $$\begin{array}{c} n_{p}∼N\left(0,\sigma_{r}^{2}+\sigma_{s}y_{p}\right), \end{array}$$

where *n*_*p*_ is noise in pixel *p* and *y*_*p*_ is the true image pixel intensity. The noise parameters *σ*_*r*_ and *σ*_*s*_ are fixed but can vary across images as sensor gain changes [15].

#### 2.3.2. Frame Displacement Model

Frame displacement directly affects how well denoising methods can take advantage of multiple frames. If the frame displacement is too large, it contributes little to denoising and may degrade performance due to misalignment. If the frame displacement is small, it is likely to contribute to the denoising performance. However, not all frame in a burst has a small displacement compared to the reference frame.

Briefly, the generated bursts need to contain frames with both large and small displacements, guiding the model to drop frames with large displacements and take advantage of frames with small displacements.

We design a frame displacement model to ensure the requests are fulfilled. Suppose *d*_*x*_, *d*_*y*_ as the horizontal and vertical displacement, respectively. They subject to a uniform distribution:

(3) $$\begin{array}{c} d_{x},d_{y}\in U\left(0,D\left(a\right)\right), \end{array}$$

where *a* is a scalar parameter and *D*(*a*) is decided by the following distribution:

(4) $$\begin{array}{c} D\left(a\right)=\left\{\begin{array}{cc} a, & \text{if }z>\frac{1}{2},\\ 16, & \text{if }z\leq \frac{1}{2}, \end{array}\right. \end{array}$$

where *z* ∈ *U*(0,1). That is, the upper limit of *d*_*x*_, *d*_*y*_ is randomly chosen between *a* and 16.

With model (4), we can control the distribution of displacement with a single displacement parameter *a*. *a* means frames are with less displacement and more similar. Since the ablation study of *a* has already been done in [12], we fix the parameter as *a*=4 in this article.

### 2.4. Model Training

Training of the proposed network is carried out in two stages. In the first stage, only the joint demosaicing and denoising module is trained, and the training method and data generation method are the same as the DRBD network in [11]. We select around 4,000 images from the Waterloo exploration dataset [16] to build clean-noisy image pairs by adding simulated noise to clean images. The type of added simulated noise can either be Gaussian or Poisson Gaussian as in (2). With the training image pairs, the proposed network can be optimized using a random gradient descent method. If blind denoising is required, the noise estimation module is also trained at this stage. The denoising module and the noise estimation module are optimized simultaneously.

When solving the blind demosaicing and denoising problem, there are two labels corresponding to the noisy raw image block *b*_*i*_: the noise-free linear RGB image block *y*_*i*_ and the noise level map *n*_*i*_. Recall that the joint demosaicing and denoising module is **dm**(·, *θ*_1_), and the noise prediction module is *G*(·, *ϕ*), and then the first stage loss function can be written as

(5) $$\begin{array}{c} L_{1}\left(b,y\right)=\min_{\theta_{1},\phi }\sum_{i}{\left\|dm\left(b_{i},\theta_{1}\right)-y_{i}\right\|}_{1}+\left(1-\lambda \right){\left\|G\left(b_{i},\phi \right)-n_{i}\right\|}_{1}, \end{array}$$

where *λ* is the scalar weight for adjusting the noise estimation and denoising weights. We take *λ*=0.75 in this article.

In the second stage, the joint demosaicing and denoising module DRBD and the multiframe denoising module MF-SE-DRDD are jointly trained. Recall that the multiframe denoising module is *F*(·, *θ*), and then, the training loss function of the multiframe denoising model is

(6) $$\begin{array}{c} L_{mf}\left(b,y,t\right)={\left\|\frac{1}{n}\sum_{i=1}^{n}f_{i}\left(dm\left(b_{i};\theta_{1}\right);\theta \right)-y\right\|}_{1}+\beta \alpha^{t}\sum_{i=1}^{n}{\left\|f_{i}\left(dm\left(b_{i};\theta_{1}\right);\theta \right)-y\right\|}_{1}, \end{array}$$

where *f*_*i*_(·; *θ*_1_) is the intermediate results before the SE module in Figure 2, namely, a part of the whole mapping *F*(·, *θ*) and *θ*_1_ ⊂ *θ*; *α* is the parameter that controls the simulated annealing rate, *β* is the initial weight of the two terms in the optimization function, and *t* represents the current iteration number of the optimization process. As *t* increases, the weight of the second term in the loss function gradually approaches zero, so that only the first term remains in the loss function.

The loss function of the second stage is the sum of the loss function of the first stage and the loss function of the multiframe denoising model:

(7) $$\begin{array}{c} L_{2}=L_{1}+L_{mf}. \end{array}$$

Under the constraint of this loss function, the noise estimation module *G*(·; *ϕ*), the single-frame joint demosaicing and denoising module **dm**(·; *θ*_1_), and the multiframe denoising module *F*(·, *θ*) are optimized at the same time.

The purpose of staged training is to avoid the network learning the two inconsistent targets of joint demosaicing and multiframe denoising at the same time without initialization, which leads to jitter in the training process and difficulty in convergence, which will be further studied in the ablation study. After training in stages, these two subtraining problems are already solved problems, and the training process is stable and easy to converge.

## 3. Experiments

To evaluate the performance of the proposed method and other comparative methods on multiframe joint demosaicing and denoising tasks, we compare the results of each method on simulated Gaussian noise and real noisy multiframe raw images and design an ablation experiment to verify effectiveness of the two-stage design.

### 3.1. Experimental Setup

#### 3.1.1. Dataset

Kodak, McM [18], and BSD [19] datasets are selected as the evaluation datasets of multiframe joint demosaicing and denoising of Gaussian noise. The noise-free images from the datasets are used as groundtruth. The input simulated multiframe Gaussian noisy images are generated by the training data generation method in this article. When experimenting with multiple frames of real raw images, this section selects the HDR+ [20] dataset taken by Google mobile phones.

#### 3.1.2. Compared Methods

Several comparison methods are selected. One is the combination of classic methods: Bayer interpolation [21] followed by V-BM4D [22]. This method first performs Bayer interpolation on each frame of raw image to complete the demosaicing, turning the problem into a multiframe denoising problem; then, the classic V-BM4D method is used to perform multiframe denoising. The combination of these two classic methods is an important reference for measuring the performance of multiframe demosaicing and denoising. The second method for comparison is BDNet [23] from ECCV 2020. This method develops an alternating learning scheme to learn to align adjacent frames and to denoise static frames separately, and applies the learned model to real-world dynamic sequences. The third is a paper method of ICCV19, referred to as M2M [24]. This method first performs single-frame joint demosaicing and denoising for each frame of raw image based on DeepJoint [1], and then, an unsupervised adjustment is performed through iterative optimization to obtain the joint demosaicing and denoising result.

#### 3.1.3. Evaluation Metrics

The groundtruth of the Gaussian noise experiment can be obtained. The peak signal-to-noise ratio (PSNR) and the structural similarity factor (SSIM) are used as evaluation metrics. For the denoising results of real multiframe raw images, this section demonstrates the superiority of the proposed method through qualitative analysis and comparison of image details.

### 3.2. Quantitative Comparison on Simulated Multiframe Raw Images

Table 1 uses source code experiments to compare Bayer interpolation [21]+V-BM4D [22], BDNet [23], M2M [24], and the proposed TwoStage method on three datasets. The input is three frames of the simulated Gaussian noise Bayer image, and the displacement parameter is *a*=4.

With a total of 9 test groups with three datasets and three noise levels, the proposed method has achieved 8 firsts, and the performance improvement increases with the raise of the noise level. When the noise level is *σ*=25, the proposed method has an average PSNR improvement of more than 0.8 dB on the Kodak and BSD500 datasets compared to the second place method M2M, and the average improvement of SSIM reaches more than 0.038. On the McM dataset, the average performance of the proposed method is weaker than M2M at *σ*=5 and is close to the average performance of M2M at *σ*=15, and the proposed method is still obvious when *σ*=25. The performance advantage shows that the proposed method has the first average performance in most test situations of multiframe demosaicing and denoising tasks, and has a stable performance advantage.

### 3.3. Qualitative Comparison on Simulated Multiframe Raw Images

**Figure 3:**
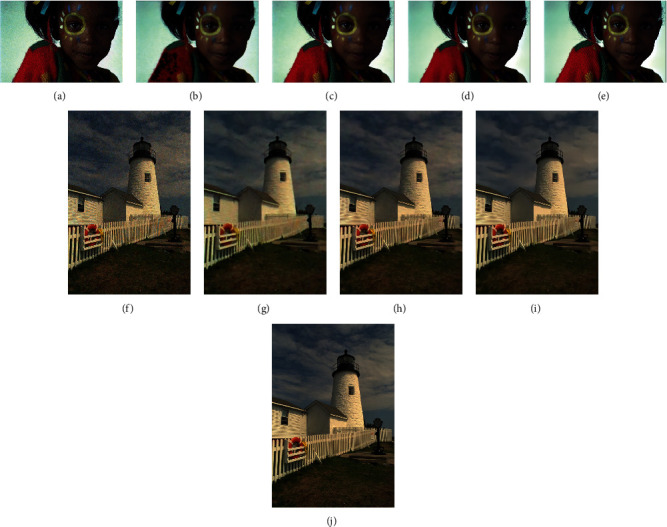
Demosaicing and denoising results on Kodak dataset when *σ*=25 (zoom in to see details). (a) Interp.+V-BM4D. (b) BDNet. (c) M2M. (d) TwoStage. (e) Groundtruth. (f) Interp.+V-BM4D. (g) BDNet. (h) M2M. (i) TwoStage. (j) Groundtruth.

This subsection shows the details of the demosaicing and denoising results of each method to illustrate its performance.  Figure 3 shows the three-frame demosaicing and denoising results of each method when the noise level on the Kodak dataset is 25. It can be seen that in (a), the Bayer interpolation + V-BM4D method cannot effectively remove the noise. The reason may be that the Bayer interpolation changes the noise distribution, which interferes with V-BM4D. The results of BDNet in (b) are blurry with black holes at the left shoulder of the girl. The results of the M2M method in (c) have many flaws in detail. For example, the girl in the picture has more obvious flaws on the face and the edge of the jaw, which affects the visual effect of the image. The results of the proposed method in (d) have a better visual effect. For example, the girl's face in the picture has no obvious artifacts and the edges are flat. The disadvantage of the proposed method is that the details of the sweater at the girl's right shoulder are not as good as M2M. (e) is the groundtruth of the scene.

Figures (f)–(j) of 3 show the results of another scene on the Kodak dataset. (f) shows that the Bayer interpolation + V-BM4D method has a poor denoising effect, and the Moiré effect is obvious at the dense fence. (g) shows that BDNet still generates blurry results with some Moiré effect at the dense fence. (h) shows the result of the M2M method has a better demosaicing and denoising effect, with more grass details retained, and there is almost no Moiré effect in dense fence areas. (i) shows that results of TwoStage have less false color than those of M2M at the cloud parts. (j) is the groundtruth of the scene.

**Figure 4:**
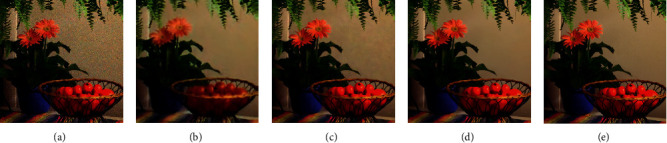
Demosaicing and denoising results on McM dataset when *σ*=25 (zoom in to see details). (a) Interp.+V-BM4D. (b) BDNet. (c) M2M. (d) TwoStage. (e) Groundtruth.

Figure 4 shows the three-frame demosaicing denoising result when the noise level is 25 on the McM dataset. Bayer interpolation with the V-BM4D method in (a) has a poor denoising effect. The result of BDNet in (b) is blurry with slight changes in brightness. Although the M2M method in (c) removes most of the noise, it leaves more obvious denoising artifacts on flat surfaces such as walls, which affects the visual effect. The proposed method in (d) effectively removes noise, and there is no residual noise on flat areas such as walls, and the visual effect is the best. (e) is the groundtruth of the scene.

**Figure 5:**
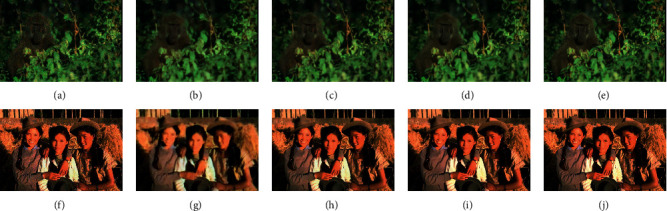
Demosaicing and denoising results on BSD500 dataset when *σ*=25 (zoom in to see details). (a) Interp.+V-BM4D. (b) BDNet. (c) M2M. (d) TwoStage. (e) Groundtruth. (f) Interp.+V-BM4D. (g) BDNet. (h) M2M. (i) TwoStage. (j) Groundtruth.

Figure 5 shows the three-frame demosaicing denoising result when the noise level is 25 on the BSD500 dataset. Bayer interpolation with the V-BM4D method in (a) cannot effectively remove noise. The result of BDNet in (b) is blurry; the M2M method in (c) leaves more noise residues and burrs at the edges of the apes. The proposed method in (d) has a better denoising effect and fewer defects. (e) is the groundtruth of the scene.

Figures (f)-(j) of 5 show the results of another scenario on the BSD500 dataset. Bayer interpolation with the V-BM4D method in (f) has a poor denoising effect. The result of BDNet in (b) keeps to be blurry; the M2M method in (h) causes visible grid-like blemishes on the girls' faces. The proposed method in (i) has a better denoising effect with almost no visible flaws. (j) is the groundtruth of the scene.

Through the qualitative analysis in this section, it can be seen that the traditional Bayer interpolation with the V-BM4D combination cannot effectively deal with the multiframe demosaicing and denoising problem, and its denoising effect is poor; BDNet is not good at aligning frames in the test case, which generates blurry results with some artifacts; the M2M method can effectively suppress the Moiré problem during demosaicing, and it is easy to leave denoising artifacts in flat areas, but more details can be preserved in some areas; the proposed method can also effectively suppress Moiré, with good visual effect and no artifacts.

### 3.4. Qualitative Comparison on Real Multiframe Raw Images

This subsection illustrates the performance of each method in processing real multiframe raw images through a qualitative comparison of denoising details. The noise estimation module of the proposed method can obtain the noise level maps of scenes, and then, the average noise level is calculated as input for the other two comparison methods.

The test multiframe images come from the HDR + dataset. The original data are in dng format. First, we use the open-source tool DCRAW1 to convert the dng format to tiff format. Raw image pixels change from 16 bits to 8 bits during the conversion process. There is no other change to the pixels value of the raw images.

**Figure 6:**
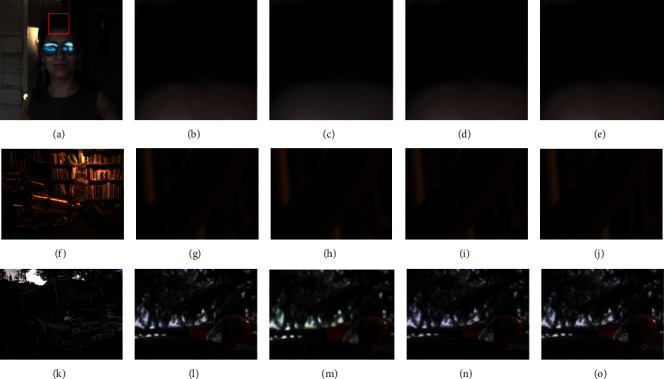
Results on real burst II (zoom in to see details). (a) A Scene. (b) Interp.+V-BM4D. (c) BDNet. (d) M2M. (e) TwoStage. (f) A Scene. (g) Interp.+V-BM4D. (h) BDNet. (i) M2M. (j) TwoStage. (k) A Scene. (l) Interp.+V-BM4D. (m) BDNet. (n) M2M. (o) TwoStage.

Figures 1 and 6 show the demosaicing and denoising results of several groups of real multiframe raw images with a sequence length of 3.  Figure 1(a) is a larger view of a TwoStage result. (b), (c), (d), and (e) are enlarged parts of the results of the three compared methods. We can find that the grid details in (b) are fuzzy and invisible; grid details in (c) are much worse than (b); the grid details in (d) are better than those in (b), but it is still unclear. The grid details in (e) are kept relatively complete. Therefore, the proposed method maintains the best detail retention ability in this test scenario.

Figure 6(a) shows a photograph of a woman. (b), (c), (d), and (e) are enlarged parts of the results of the three compared methods. The result of the combination of traditional methods in (b) has faint horizontal light and dark stripes. The results of BDNet in (c) are smoothy with slight changes in brightness. The result of M2M in (d) suffers from obvious grid-like defects in the woman's forehead and the rear wooden. The result of the proposed method in (e) is relatively good without defects. Figures 6(f)–6(j) shows an indoor scene. The result of V-BM4D in (g) contains noise. The result of BDNet in (h) is noise-free but loses details of the curved text on the wheel. The curved text on the wheel in the result of M2M in (i) is blurred. In comparison, our result in (j) is noise-free while the same text is clearer.

Figures 6(k)–6(o) shows an outdoor scene. The tree branches details of the BDNet results in (m) are lost. The tree branches part of the M2M result in (n) is blurry; in comparison, the result of V-BM4D in (l) and the proposed method in (o) looks clear.

In summary, V-BM4D cannot remove noise effectively; BDNet can remove noise but turns to generate blurry results; the results of M2M is less blurry; our proposed TwoStage method generates results with the best visual quality on real mutliframe raw images in the test.

### 3.5. Ablation Study

In the multiframe demosaicing and denoising experiment, the proposed model is mainly composed of DRDD responsible for single-frame joint demosaicing and denoising and MF-SE-DRDD responsible for multiframe denoising. The ablation experiment will test the following: (1) replacing DRDD in the model with the classic Bayer interpolation method to explore its necessity; (2) using DRDD to directly do single-frame joint demosaicing and denoising to explore the necessity of multiframe denoising; and (3) comparing one-stage with two-stage training to demonstrate the effect of two-stage training.

Table 2 lists the results of the first ablation experiment. Replacing DRDD with the classical interpolation leads to a performance drop in 8 out of 9 test cases. Besides, the higher the noise level, the more the performance is reduced. Specifically, when *σ*=25 on the Kodak dataset, the average performance degradation of PSNR reaches 1.05 dB, and the average performance degradation of SSIM reaches 0.0224. This shows that DRDD plays an important role in the method, especially in the large noise condition.

Comparing the proposed method with the single-frame denoising method DRDD in the second experiment, it can be found that when *σ*=25, there is a consistent performance improvement on different datasets; when the noise level *σ*=15, there is a slight performance gain in Kodak datasets and a slight performance decrease on the remaining datasets. This shows that the multiframe method has a better performance improvement when the noise level is large, and the single-frame method is more appropriate when the noise level is small.

**Figure 7:**
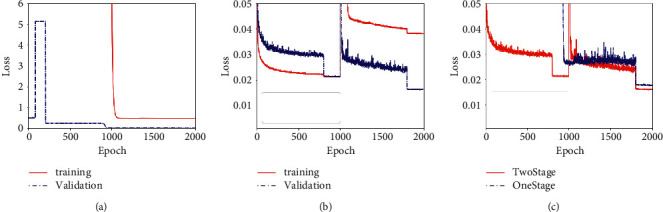
Comparison of training and validation loss of one-stage training with two-stage training. (a) One-stage. (b) Two-stage. (c) Comparison of validation losses.

In the third experiment, we conduct a one-stage training experiment in comparison with the two-stage one. Recall that the total number of training process is 2,000 epoches, and each stage takes 1,000 epoches. The comparison one-stage experiment uses the same training settings. It can be found in Figure 7(a) that one-stage training takes around 900 epoches before the training and validation loss reach a reasonable value range. In comparison, the two-stage training can keep the training and validation loss in a low value range during the whole training process in Figure 7(b). The validation losses of one-stage and two-stage training are plotted in the same figure in Figure 7(c). It is clear that the validation loss of two-stage training converges more quickly and to a lower level, which suggests the two-stage training is a better policy.

### 3.6. Running Time

Table 3 lists the average running time on three frames. Among them, interpolation + V-BM4D uses MATLAB to run on CPU, and M2M and TwoStage use the Pytorch framework to run on GPU. Running time data are measured on a desktop computer with Intel I7–5390K CPU and Nvidia GTX 1080Ti GPU.

The first method and the latter two methods do not run on the same device, and its running time is only listed for reference.

It can be seen that the proposed TwoStage has an advantage over M2M in speed. This is because M2M is iteratively optimized, and it takes more time even on the GPU. The proposed method only needs to run model inference and is much faster. BDNet is the fastest method.

## 4. Conclusion

A two-stage demosaicing and denoising method for burst images is proposed. The basic idea is to do joint demosaicing and denoising on single frames first, and then to do multiframe denoising on the initial results. In this process, each frame of the input image undergoes single-frame and multiframe denoising in two stages, contributing to the final denoised linear RGB image. For a network design, this article proposes a two-stage training method to ensure that the model converges to a good solution. Experiments on multiframe Bayer images with simulated Gaussian noise show that the proposed method has obvious performance advantages and speed advantages compared with similar methods. Experiments on actual multiframe Bayer images verify the denoising effect and detail retention ability of the proposed method. Ablation study shows the effectiveness of each CNN module.

## Figures and Tables

**Table 1:** Comparison of multiframe joint demosaicing and denoising results (*a*=4).

Dataset	Noise level	BDNet		Interp.+V-BM4D		M2M		TwoStage	
		PSNR	SSIM	PSNR	SSIM	PSNR	SSIM	PSNR	SSIM

Kodak	*σ*=5	20.29	0.513	33.05	0.9213	35.90	0.9429	**36.33**	**0.9487**
	*σ*=15	19.47	0.4789	28.63	0.7548	31.45	0.8623	**31.88**	**0.8774**
	*σ*=25	20.49	0.5107	25.76	0.6161	28.90	0.7783	**29.77**	**0.8198**

McM	*σ*=5	16.57	0.4417	32.99	0.9010	**35.24**	**0.9360**	34.92	0.9284
	*σ*=15	18.82	0.4995	28.98	0.7630	31.40	0.8713	**31.53**	**0.8732**
	*σ*=25	17.37	0.4564	26.16	0.6436	29.01	0.7941	**29.67**	**0.8297**

BSD 500	*σ*=5	19.02	0.4492	32.80	0.9310	34.98	0.9524	**35.41**	**0.9544**
	*σ*=15	18.88	0.4389	28.36	0.7845	30.47	0.8689	**30.92**	**0.8805**
	*σ*=25	19.12	0.4432	25.53	0.6585	27.95	0.7799	**28.75**	**0.8183**

**Table 2:** Ablation study (PSNR/SSIM, *a*=4).

Dataset	*σ*	Interpolation + MF-SE-DRDD	DRDD	TwoStage
Kodak	5	36.10/0.9472	36.15/0.9464	**36.33**/**0.9487**
	15	31.13/0.8656	31.61/0.8712	**31.88**/**0.8774**
	25	28.72/0.7975	29.48/0.8101	**29.77**/**0.8198**

McM	5	34.67/0.9276	**35.17**/**0.9312**	34.92/0.9284
	15	30.85/0.8544	**31.54**/0.8708	31.53/**0.8732**
	25	28.35/0.7896	29.47/0.8199	**29.67**/**0.8297**

BSD 500	5	35.51/0.9551	**35.43**/0.9533	35.41/**0.9544**
	15	30.41/0.8710	30.70/0.8751	**30.92**/**0.8805**
	25	27.80/0.7947	28.44/0.8084	**28.75**/**0.8183**

**Table 3:** Running time (seconds).

Dataset	Interp.+V-BM4D (CPU)	M2M(GPU)	TwoStage(GPU)	BDNet(GPU)
Kodak	6.43	17.68	0.44	0.24
McM	4.74	11.89	0.26	0.11
BSD500	2.47	8.03	0.17	0.05

## Data Availability

The datasets Kodak, McM, BSD500, and HDR + are publicly available: (1) Kodak at http://www.cs.albany.edu/∼xypan/research/snr/Kodak.html,(2) McM at http://www4.comp.polyu.edu.hk/∼cslzhang/CDM_Dataset.html,(3) BSD500 at https://www2.eecs.berkeley.edu/Research/Projects/CS/vision/bsds/, and(4) HDR + at http://www.hdrplusdata.org/dataset.html.
